# Ras functional proximity proteomics establishes mTORC2 as new direct ras effector

**DOI:** 10.18632/oncotarget.27025

**Published:** 2019-08-27

**Authors:** Joanna R. Kovalski, Ronald L. Shanderson, Paul A. Khavari

**Affiliations:** ^1^ Program in Epithelial Biology, Stanford University, Stanford, CA 94305, USA; ^2^ Program in Cancer Biology, Stanford University, Stanford, CA 94305, USA; ^3^ VA Palo Alto Healthcare System, Palo Alto, CA 94304, USA

**Keywords:** Ras, BioID, proteomics, CRISPR, mTORC2

## Abstract

Although oncogenic mutations in the three major Ras isoforms, *KRAS*, *HRAS* and *NRAS*, are present in nearly a third of human cancers, therapeutic targeting of Ras remains a challenge due to its structure and complex regulation. However, an in-depth examination of the protein interactome of oncogenic Ras may provide new insights into key regulators, effectors and other mediators of its tumorigenic functions. Previous proteomic analyses have been limited by experimental tools that fail to capture the dynamic, transient nature of Ras cellular interactions. Therefore, in a recent study, we integrated proximity-dependent biotin labeling (BioID) proteomics with CRISPR screening of identified proteins to identify Ras proximal proteins required for Ras-dependent cancer cell growth. Oncogenic Ras was proximal to proteins involved in unexpected biological processes, such as vesicular trafficking and solute transport. Critically, we identified a direct, *bona fide* interaction between active Ras and the mTOR Complex 2 (mTORC2) that stimulated mTORC2 kinase activity. The oncogenic Ras-mTORC2 interaction resulted in a downstream pro-proliferative transcriptional program and promoted Ras-dependent tumor growth *in vivo*. Here we provide additional insight into the Ras isoform-specific protein interactomes, highlighting new opportunities for unique tumor-type therapies. Finally, we discuss the active Ras-mTORC2 interaction in detail, providing a more complete understanding of the direct relationship between Ras and mTORC2. Collectively, our findings support a model wherein Ras integrates an expanded array of pro-oncogenic signals to drive tumorigenic processes, including action on mTORC2 as a direct effector of Ras-driven proliferative signals.

## INTRODUCTION

Ras GTPases are molecular switches that integrate external growth factor signaling with internal biochemical changes. Wildtype Ras is tightly regulated to maintain an equilibrium between the guanosine diphosphate(GDP)-bound inactive state and the guanosine triphosphate(GTP)-bound active state that is disrupted by mutations in *RAS* [1]*.* Mutations in the three major Ras isoforms, *HRAS*, *NRAS*, and *KRAS*, which impair GTPase activity and/or the ability of Ras to interact with negative regulators are found in approximately a third of human cancers [2]. Despite the role of Ras as a nexus in cancer signaling, the strength of the Ras-GTP interaction and lack of targetable pockets has precluded broadly applicable therapeutic intervention. Thus, the effectors and regulators of Ras are high priority drug targets. The complexity of Ras regulation and signaling, however, present considerable current challenges [3]. A deeper understanding of the intricate mechanisms of Ras regulation and downstream signaling will be key to successfully targeting oncogenic Ras to treat tumors. Therefore, determining the full spectrum of Ras interactors is a critical endeavor.

### Proteomics identifies novel Ras interactors

Our current understanding of the Ras interactome stems principally from affinity purification approaches. From these affinity-based approaches, such as co-immunoprecipitation, critical and well-known components of Ras signaling such as PI3K and the Raf family of serine/threonine kinases were identified [4, 5]. More recently studies have added unbiased mass spectrometry analysis after the affinity purification (AP-MS) to more fully capture the Ras protein-protein interactome [6]. While capable of identifying strong interactions, AP-MS biases against transient and low-affinity interactions, in particular those that rely on lipid membranes for stability [7, 8]. Proximity-dependent biotin labeling (BioID) is a powerful technology to enable *in situ* identification of Ras interactors. BioID relies on the fusion of a protein of interest, such as Ras, with a mutant *Escherichia coli* biotin ligase (birA*) that is capable of biotinylating lysines within an ~10 nm radius [9]. Furthermore, BioID is carried out in living cells, allowing for *in situ* identification of Ras interactors while maintaining thorough lysis and stringent purification conditions [10]. Additionally, BioID enables the identification of interactions between proteins that are stabilized by lipid membranes or other cellular compartments, which are destroyed during cell lysis in AP-MS [11]. Therefore, BioID can provide new insights for membrane-bound proteins such as Ras.

We performed BioID with both the wild-type and mutant Ras proteins for the three main isoforms of Ras in the cancer cell type where each isoform is frequently mutated [12]. Our experiments yielded 690 Ras-proximal proteins, with 150 of these interactors being common among the 3 Ras isoforms. These common proximal proteins spanned several functional classifications. The identification of proteins involved in cytoskeletal function, adhesion junctions, and phosphorylation signaling cascades confirmed current models of Ras function and regulation [13]. Some novel functional groups proximal to all Ras isoforms include vesicular transport proteins, small molecule transporters, and proteins involved in transmembrane signaling that are not directly related to receptor tyrosine kinase signaling. This dataset provides the Ras biology community with additional candidates for testing hypotheses related to Ras function and regulation.

### Isoform specific interactors

**Figure 1:**
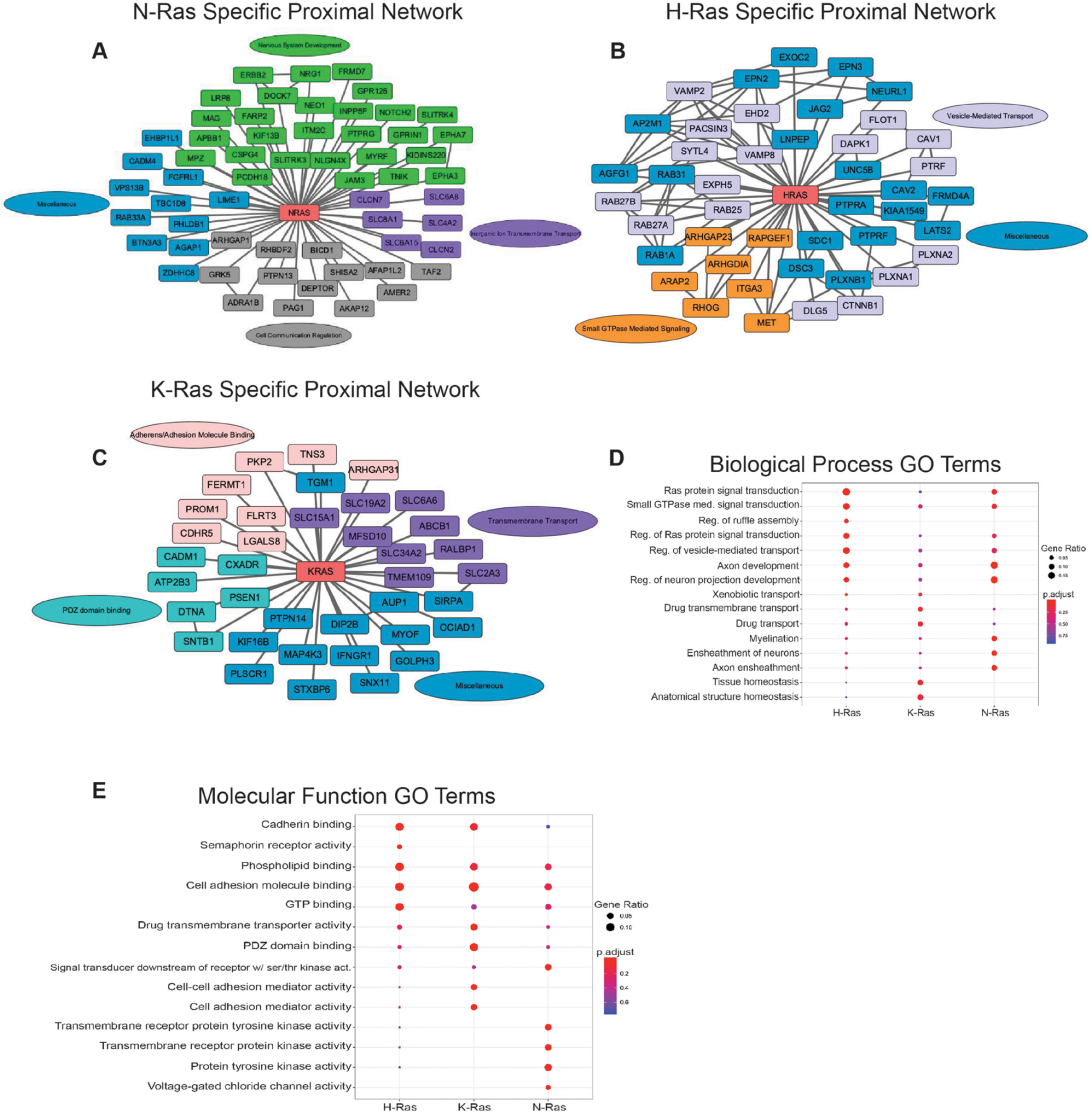
Ras isoform specific proximal proteome networks. (**A**–**C**) Ras isoform specific proximal proteome networks with selected interactors. Connections between non-Ras nodes are defined as known interactors having experimental/biochemical data of the interaction, interaction between putative homologues, or association in STRING curated databases. Proximal Ras interactors colored by Gene Ontology (GO) terms. (**D**) Enriched biological process GO terms for each of the 3 Ras isoform specific proximal interactomes. (**E**) Enriched molecular function GO terms for the 3 Ras isoform unique proximal proteomes. Size of circles (Gene Ratio) indicates the fraction of proteins within the interactome specific to that GO term. Benjamini-Hochberg adjusted *p*-value.

Here, we have taken advantage of the above experimental design to provide new, additional examination of proteins that are selectively proximal to each Ras isoform. The isoform-specific proximal proteomes, along with further experimentation, can elucidate both the shared and unique roles of each Ras isoform (Figure 1A–1C). The proximal interactome of N-Ras was enriched for proteins related to neurogenesis (Figure 1A and 1D), including nodes centered on ERBB2 and Ephrin receptors [14, 15], which have been linked to tumor progression [16]. Additionally, the specificity of the interactors may hint at non-overlapping receptor and Ras pairings where different Ras isoforms transduce unique signals across the membrane. Supporting this idea, H-Ras and K-Ras also interact with unique receptors such as MET and IFNGR1, respectively (Figure 1B–1C). Many of these specific interactors may also reflect the cancer type setting in which we identified the interactome of the isoforms. Overall, understanding these cancer-type and isoform specific interactors may enable highly specific and effective therapeutics opportunities.

The isoform-specific proximal protein interactome data also suggest a role for K-Ras in broad plasma membrane (PM) organization. The K-Ras interactome is enriched for PDZ domain binding proteins and transmembrane transporters (Figure 1C and 1E). As a mediator of protein-protein interactions, the PDZ-domain is particularly interesting in the context of K-Ras, which can form nanoclusters on the PM [17]. This suggests a model wherein K-Ras nanoclusters act as nucleating proteins for signaling complexes and cytoskeletal structures. This extends a paradigm from previous work demonstrating that a PDZ-domain containing protein, which is responsible for organizing adherens junctions, AFDN, interacts with Ras to link it to cellular junctions [18]. In conjunction with the enrichment of the K-Ras interactome for cell adhesion proteins, such as SNTB1 and DTNA, which are responsible for clustering receptors, these data expand the myriad ways in which K-Ras reorganizes the plasma membrane and cytoskeleton in response to proliferative signals [19]. This is further confirmed by the enrichment of GO terms for cell-cell adhesion mediator activity and cell adhesion mediator activity in the K-Ras interactome (Figure 1E). Interestingly, H-Ras interacts with an extensive network of Rab GTPases, SNARES, and associated proteins (Figure 1B). This suggests that H-Ras is extensively trafficked throughout the cell with a large endomembrane distribution and/or is an important regulator of vesicular transport in conjunction with Rab GTPases. This dataset provides starting points for future experiments designed to understand how cancers of varying origins rely on the tumorigenic properties of specific Ras isoforms and why certain cancers are sensitive to perturbations in some pathways, but not others. Illuminating the differential role that each Ras isoform plays in transformation and how these novel isoform-specific interactions in tumor cells intersect with normal Ras signaling will elucidate the mechanisms underlying tissue-specific mutations of Ras isoforms in cancer. Ultimately, this knowledge will enable new treatments targeting a specific mutant Ras isoform and the processes it regulates.

### Critical interactors for Ras-dependent proliferation

Though proximity proteomics identifies proteins in the Ras milieu with high confidence, we cannot discern their functional impacts in cancer. To identify which interactors are critical for Ras-driven proliferation, we performed a CRISPR knockout screen in Ras-dependent and Ras-independent cancer cell lines [12]. By combining our proteomic and genetic approaches, we identified several Ras interactors that were preferentially proximal to oncogenic Ras that also decreased the growth of Ras-dependent cancer cells. These functional interactors fell into both known and novel signaling modules. The known modules include Raf-Src signaling and PI3K signaling. For these well-known interactors, our screen verified prior experimental observations. For example, literature suggests that Raf1 plays a unique role in tumorigenesis that cannot be compensated for by A-Raf or B-Raf [20]. Our combined screens recapitulate this finding, with all 3 Raf isoforms enriched in the oncogenic Ras interactome, but Raf1 as the only significant CRISPR screen hit. The novel modules consist of transmembrane solute carriers, vesicular transport machinery, and mTOR along with the components of the mechanistic target of rapamycin complex 2 (mTORC2). From these new functional modules, we can derive some heretofore unidentified, but common functions of the oncogenic Ras isoforms in cancer.

There were several solute carrier proteins (SLCs) that were enriched via our combined proteomic and genetic analysis. In particular, SLC3A2 and its partner proteins, SLC7A5 and SLC7A11, are intriguing novel Ras proximal proteins [12]. SLC3A2 is an integrin co-receptor that serves as a heavy chain for SLC7 family members and is necessary for their function [21]. SLC3A2 heterodimerizes with SLC7A5 to form LAT1, a branched chain amino acid transporter, or with SLC7A11 to form system x_c_^−^, a cystine-glutamate antiporter [22]. All 3 components have been linked to tumorigenesis and SLC3A2 loss can be protective in Ras-driven tumor models [23–25]. While we have yet to elucidate the precise nature of the SLC3A2-Ras interaction, the data suggest tantalizing hypotheses. Interestingly, although SLC3A2 interacts equally with wild-type and oncogenic Ras, SLC7A11 is significantly enriched in the oncogenic Ras interactome and SLC7A5 is moderately so, suggesting that Ras may influence their interactions with SLC3A2. Previous research provides a precedent for Ras altering cellular metabolism through several mechanisms, including upregulation of transporter GLUT1 (SLC2A1) [26]. Performing additional BioID experiments on the SLC family of proteins in the context of Ras activation could shed light on the role of Ras in regulating this complex family of proteins. Additionally, metabolic profiling after disruption of the members of the SLC3A2 interactome would further elucidate the specific role of oncogenic Ras in regulating SLC3A2-containing complexes and expand the role of Ras in metabolism. Interaction with SLCs may be another, more direct way for Ras to alter the metabolism of cells to promote tumorigenesis.

Another functional module prominent in the data is centered on vesicular transport proteins. Understanding the mechanistic role these proteins play in Ras regulation will fill critical gaps in current knowledge about regulation of Ras localization. Our integrated proteomic and genetic screens identified VPS51 and associated protein STX6, which are involved in trafficking of early and late endosomes to the trans-golgi network, where H- and N-Ras are known to be localized [27, 28]. We also found significant enrichment of proteins in the exocyst complex, which is responsible for the golgi to plasma membrane transport of secretory vesicles. EXOC3 was necessary for Ras-dependent proliferation and preferentially proximal to mutant Ras, which is consistent with H-Ras reliance on the exocytic pathway for its localization and provides new and specific insight into the complex component that may be critical for this process [29]. Additionally, we identified several SNARE proteins. Though it is known that K-Ras requires vesicles for transportation from recycling endosomes to the plasma membrane, the particular vesicular proteins necessary for this process and the degree of their specificity are largely unknown [30]. Our data suggest that K-Ras may interact with specific SNARE proteins for K-Ras transport, likely from endosomes to the plasma membrane. Perturbing K-Ras specific transport proteins is of great clinical importance as it is the most commonly mutated of the Ras isoforms. Interestingly, we also identified a negative regulator of Ras, LZTR1, which has been implicated in congenital “RASopathies” [31] and was recently demonstrated to facilitate Ras ubiquitination, restricting Ras to endosomal compartments where it cannot actively signal [32, 33]. This demonstrates the broad utility of the integrated BioID-CRISPR screening approach and its ability to identify proximal proteins that modulate Ras under homeostatic conditions as well as pro-tumorigenic effectors and regulators of oncogenic Ras. Moreover, using the previously mentioned isoform-specific data, we may be able to specifically prevent trafficking of the driver, mutant Ras isoform to lessen or prevent toxicity associated with targeting Ras pathways more generally. A better understanding of the mechanisms of Ras transport may help in the revival of efforts to drug proteins critical for Ras trafficking to its sites of action.

The top-ranking module common to all three Ras isoforms was centered on mTOR and encompassed the components of the mechanistic target of rapamycin complex 2 (mTORC2). mTOR functions within at least two distinct complexes mTORC1 and mTORC2 with the latter regulating multiple cellular processes, such as cell survival and metabolic dysregulation through Akt [34]. Ras is known to regulate mTORC1 downstream of the MAPK and PI3K pathways by inactivating the negative regulator TSC1/2, leading to mTORC1 activity [35]. Although mTORC2 function has been shown to be directly dependent on PI3K activity [36] and Ras can bind mTORC2 component, MAPKAP1 [37, 38], no previous work implicated Ras in direct binding to mTOR or regulation of mTORC2 kinase activity. We demonstrated that mTORC2 is a new, direct downstream effector of Ras [12].

### mTORC2 is a *bona fide* Ras effector

Extremely limited prior evidence places Ras in direct proximity to mTORC2 with no known direct interaction between Ras and mTOR itself. Intriguingly, the integration of BioID proteomics and CRISPR genetic screen data demonstrated a proximal, preferential interaction between mutant Ras and mTOR itself. Co-immunoprecipitation experiments supported an interaction between oncogenic Ras and endogenous mTOR in N-Ras mutant melanoma cells. To gain insight into the contact points between mTOR and active Ras, we used crosslinking-mass spectrometry (XL-MS), enabling unbiased, yet specific detection of interactions between the particularly large mTOR protein and active Ras [12]. The XL-MS analysis identified direct contact between the kinase domain of mTOR and residues near the effector binding domain of GTP-bound Ras. This suggests a mode of interaction analogous to the direct association of mTOR with the small GTPase Rheb, in which direct binding of active Rheb to mTOR, potentially through the mTOR kinase domain, allosterically promotes mTORC1 kinase function [39, 40]. Indeed, active, Ras-GTP bound the mTOR kinase domain with a greater affinity than GDP-bound Ras in quantitative microscale thermophoresis (MST) biophysical measurements. Mutagenesis of the Ras effector binding domain altered mutant Ras proximity to mTOR, further substantiating mTOR as a classical Ras effector. Finally, illustrating relevance to human cancer, mTOR and Ras proximity was significantly increased in K-Ras mutant colon adenocarcinoma specimens as compared to K-Ras wild-type tumors. Therefore, GTP-bound Ras, as in the oncogenic form, is competent to bind directly to mTOR via the kinase domain.

Although mTOR participates in at least two distinct complexes, mTORC1 and mTORC2, our analysis identified mutant Ras proximity with mTORC2 components only. Subsequent experiments demonstrated that the mTORC2 components, Rictor and MAPKAP1, preferentially co-immunoprecipitated with oncogenic compared to wild-type Ras and were required for mutant Ras and mTOR proximity in mutant N-Ras melanoma cells. Mutagenesis of the Ras effector binding domain also altered oncogenic Ras proximity to MAPKAP1 and Rictor, further solidifying that the mTOR-Ras interaction occurs in the context of mTORC2. If mTORC2 is a *bona fide* Ras effector, GTP-bound Ras should alter the functional activity of mTORC2 in phosphorylating its substrates. To test this, we employed both *in vitro* kinase assays as well as in cell measurements of endogenous mTORC2 activity. First, mTORC2 immunoprecipitated from cells exhibited increased kinase activity towards an Akt tail substrate when co-expressed with mutant Ras as well as in the presence of recombinant GTP-loaded Ras protein. Together, these experiments suggest that Ras binds mTORC2 directly to promote structural changes and increase mTORC2 phosphorylation activity, analogous to the impact of active Ras on Raf1 or PI3K [41, 42]. Furthermore, an in-cell kinase assay where the mTORC2 substrate, Akt, can be recruited to specific subcellular compartments [11] showed that mTORC2 activity at the plasma membrane is dependent upon mutant Ras. Taken together, the quantitative direct binding data and the functional kinase assays indicate that mTORC2 is a new, direct Ras effector.

### Disrupting the Ras-mTORC2 interaction impedes tumorigenesis

As a novel direct effector of oncogenic Ras, mTORC2 provides an attractive therapeutic target. All existing chemical inhibitors of mTORC2 also inhibit the activity of mTORC1, blocking the shared ATP binding site of mTOR. Therefore, we sought an approach that specifically disrupted mTORC2 function while leaving mTORC1 unaltered. Previous research has implicated MAPKAP1 as a direct Ras binding protein via a putative Ras binding domain (RBD) [37] and a membrane binding protein via its PH domain [43]. Our crosslinking mass spectrometry and MST direct binding experiments definitively and quantitatively demonstrated that Ras-GTP bound the previously posited RBD of MAPKAP1, particularly near three key residues with high homology to the Raf1 RBD [12, 44]. Therefore, altering MAPKAP1 may disrupt mTORC2 plasma membrane localization and its interaction with Ras, which, as we demonstrated, are both required for full mTORC2 kinase activity.

Given the paucity of mTORC2-specific chemical inhibitors, we employed a peptide-based approach. Specifically, a minimal 192 amino-terminal fragment of MAPKAP1 that is sufficient to maintain the integrity of the complex but lacks the RBD and PH domains [45]. Expressing this minimal MAPKAP1 fragment decreased mutant Ras and mTOR proximity and impeded mTORC2 kinase activity at the plasma membrane. Expression of the MAPKAP1 deletion decreased cell cycle and DNA replication gene expression in mutant Ras-dependent cancer cell lines, mirroring the transcripts specifically decreased when we knocked down either *RICTOR* or *MAPKAP1* in the same cancer cells. The MAPKAP1 deletion associated gene signature was greatly enriched in N-Ras wild-type melanoma patient data as compared to N-Ras mutant tumors, consistent with the premise that a portion of N-Ras impacts are mediated through mTORC2 components. Finally, we assayed the ability of the MAPKAP1 deletion to impede *in vivo* tumorigenesis of oncogenic Ras-dependent cancer cells. Critically, expression of the MAPKAP1 deletion hindered the subcutaneous growth of H-Ras mutant bladder cancer, K-Ras mutant colon cancer and N-Ras mutant melanoma cancer cells, but not tumorigenesis of cancer tissue type-matched wild-type Ras cells. The tumors expressing the MAPKAP1 deletion displayed decreased phosphorylation of mTORC2 substrates with minimal impacts on parallel pathways, such as MAPK and mTORC1 signaling. Our data show that the oncogenic Ras-mTORC2 interaction is required for Ras-dependent tumorigenesis and suggest a new therapeutic opportunity for difficult-to-drug mutant Ras cancers.

## CONCLUSIONS AND FUTURE PERSPECTIVES

The data generated by the combined BioID proteomics and CRISPR genetics screening effort above represent a new resource for Ras studies, expanding the proximal proteome of all three major Ras isoforms and specifically identifying interactors critical to Ras-dependent cancer cell growth. These data catalyzed efforts that demonstrated that active Ras can bind directly to mTORC2 through both mTOR and MAPKAP1 and that this interaction regulates mTORC2 kinase activity specifically at the plasma membrane, thereby establishing that mTORC2 is a new direct Ras effector. Disrupting the mTORC2 and oncogenic Ras interaction with a minimal fragment of MAPKAP1 dysregulated cell cycle and pro-proliferation gene expression, and impeded Ras-dependent *in vivo* tumorigenesis, underscoring the key role of mTORC2 as a Ras effector and illustrating the therapeutic potential of mTORC2 targeting in Ras-driven cancers.

This work prompts new biological questions that will form the basis of future studies to understand and target Ras. A key question to resolve for effective and specific therapeutic targeting is how the Ras-mTORC2 interaction results in transcriptomic changes, particularly the decreased expression of central cell cycle regulators. Additional studies are warranted to decipher the precise mTORC2 substrates impacted by oncogenic Ras activation, including potential novel substrates, as well as any accessory proteins that might tune mTORC2 function or specify substrate selection. In particular, BioID analysis of mTORC2 with or without active Ras signaling may illuminate candidate modifiers, transmitters or intermediaries of the Ras-mTORC2 signal. Additionally, phosphoproteomic analyses can shed light on the precise signaling cascades activated between Ras-mTORC2 contact and the transcriptional response. Another point of inquiry is the differential role of the manifold MAPKAP1 isoforms in Ras stimulation of mTORC2. MAPKAP1 exists as at least five isoforms [46] that contain variable portions of the Ras binding domain and the PH domain. Thus, mTORC2 may localize differently and bind to Ras less avidly depending upon the incorporated MAPKAP1 isoform. Intriguingly, all isoforms maintain the three critical MAPKAP1 residues required for Ras binding, suggesting an obligate relationship between active Ras and MAPKAP1, although the interaction may also occur outside the context of mTORC2. However, some MAPKAP1 isoforms with partial RBDs may not be competent to fully bind Ras, resulting in mTORC2 molecules with differential sensitivity and responsiveness to Ras signaling. The identification of mTORC2 as a *bona fide* direct effector of Ras provides a new avenue of study with substantial therapeutic implications.

**Figure 2:**
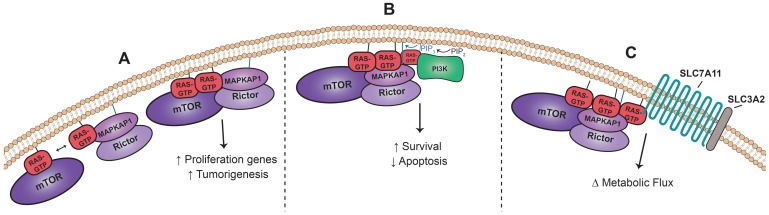
Ras-mTORC2 Interaction Coordinates Signaling. (**A**) Ras is competent to bind to both mTOR and MAPKAP1 directly and may dimerize to facilitate mTORC2 formation and/or recruitment and maintenance at the plasma membrane in addition to mTORC2 activation. MAPKAP1 and Rictor form a stable complex independent of mTOR [38]. (**B**) Ras nanoclusters may enable complete mTORC2 activation via physical proximity to PI3K. PIP_3_ produced by PI3K binds to MAPKAP1, opening the mTORC2 active site for substrate entry [43]. (**C**) Ras nanoclusters may bring mTORC2 into physical proximity with its substrate substrate SLC7A11 [52] part of a cysteine-glutamate antiporter, which is also present in the functional oncogenic Ras interactome.

Placing the finding of mTORC2 as a direct Ras effector into the broader Ras interactome establishes a cohesive model wherein Ras is a predominant signaling nexus that physically couples and integrates an even more extensive array of pro-oncogenic signals and processes than previously understood. In the support of Ras as an integrator of diverse cellular processes, several studies have shown that Ras can form dimers and even higher order nanoclusters, which are required for complete downstream pathway activation [47–49]. A prime example is the Ras-enabled dimerization of Raf1, which is necessary for downstream MAPK pathway activation [50, 51]. With respect to the mTORC2-Ras interaction, Ras dimerization may facilitate the formation and/or recruitment of mTORC2 at the plasma membrane, in addition to activating mTORC2 (Figure 2A). Indeed, the higher affinity of active Ras for MAPKAP1 than mTOR suggests that Ras might participate in the stepwise assembly and maintenance of the complex with MAPKAP1 bringing along Rictor and mTOR [38]. Once Ras and mTOR are co-localized at the lipid membrane, physical proximity may further promote enhanced affinity between Ras-GTP and mTOR, in part through its kinase domain, to stimulate full mTORC2 activity. Further structural studies of Ras-GTP with mTORC2 and/or mTOR or MAPKAP1 alone may shed light on the precise nature of the interactions. Moreover, Ras may also promote the physical proximity of several distinct protein complexes on or near the plasma membrane. Previous work suggests that PI3K’s production of phosphatidylinositol(3,4,5)-trisphosphate (PIP_3_) is required for complete mTORC2 kinase activity [43]. This model holds that the MAPKAP1 PH domain binds to PIP_3_, opening the “lid” blocking the active site of mTORC2 and, thereby, allowing substrate access for phosphorylation. We propose that additional individual Ras-GTP molecules can bind PI3K or mTORC2 and through Ras dimerization or nanoclustering bring PI3K and mTORC2 into physical proximity (Figure 2B). This proximity should promote and enhance full mTORC2 kinase activity by placing mTORC2 directly next to the source of PIP_3_. Through these models, we suggest that Ras carries out multiple functions while bound to its effectors, serving to nucleate interdependent signaling complexes as well as physically promoting effector activity.

Finally, Ras nanoclustering may also serve as a nexus to bridge two of the new functional oncogenic Ras network modules. A recent study demonstrated that mTORC2 directly phosphorylates system x_c_^−^ component, SLC7A11, decreasing antiporter function and modulating the amino acid balance within the cell [52]. Given the increase in oncogenic Ras proximity to both mTORC2 and SLC7A11, we hypothesize that active Ras might serve to bring these two key complexes into proximity, integrating growth factor signaling with amino acid metabolism in a cancer setting (Figure 2C). The oncogenic Ras proximal protein interactome highlights the highly dynamic and pervasive role of active Ras in physically and functionally linking and regulating a diverse set of cellular processes and pathways. Thus, providing ample hypotheses for future experimental studies and hopefully impactful new clinical targets.

The *in vivo* tumorigenesis studies with the MAPKAP1 deletion support the therapeutic potential of disrupting the mutant Ras and mTORC2 interaction. Future experiments to examine the anti-tumorigenic impact of progressively shorter MAPKAP1 fragments may enable the development of a peptide-based therapeutic strategy. Additionally, the reverse tactic of a MAPKAP1 peptide containing the minimal Ras binding domain, as derived from MST experiments, may serve to efficiently compete mutant Ras away from mTORC2, as has been done for Raf1 [53, 54]. However, a more conventional small molecule mTORC2-specific inhibitor may prove to be a more fruitful approach and enable easier translation to the clinic. In fact, a recent study screened a compound library for the ability to disrupt mTORC2 complex formation and found a hit that selectively inhibited Rictor-mTOR association, supporting the feasibility of this strategy [55]. Our integrated proteomic and genetic analysis provides a framework for future studies of hard to drug oncogenes or other poorly understood mediators of human disease and demonstrates the power of this approach to derive novel therapeutic opportunities for unmet clinical needs.
